# Artificial Intelligence–Based Traditional Chinese Medicine Assistive Diagnostic System: Validation Study

**DOI:** 10.2196/17608

**Published:** 2020-06-15

**Authors:** Hong Zhang, Wandong Ni, Jing Li, Jiajun Zhang

**Affiliations:** 1 Computer Center Guanganmen Hospital China Academy of Chinese Medical Sciences Beijing China; 2 Certification Center of Traditional Chinese Medicine Physician Qualification State Administration of Traditional Chinese Medicine Beijing China; 3 Department of Software Engineering NCT Lab Corp Billerica, MA United States

**Keywords:** traditional Chinese medicine, TCM, disease diagnosis, syndrome prediction, syndrome differentiation, natural language processing, NLP, artificial intelligence, AI, assistive diagnostic system, convolutional neural network, CNN, machine learning, ML, BiLSTM-CRF

## Abstract

**Background:**

Artificial intelligence–based assistive diagnostic systems imitate the deductive reasoning process of a human physician in biomedical disease diagnosis and treatment decision making. While impressive progress in this area has been reported, most of the reported successes are applications of artificial intelligence in Western medicine. The application of artificial intelligence in traditional Chinese medicine has lagged mainly because traditional Chinese medicine practitioners need to perform syndrome differentiation as well as biomedical disease diagnosis before a treatment decision can be made. Syndrome, a concept unique to traditional Chinese medicine, is an abstraction of a variety of signs and symptoms. The fact that the relationship between diseases and syndromes is not one-to-one but rather many-to-many makes it very challenging for a machine to perform syndrome predictions. So far, only a handful of artificial intelligence–based assistive traditional Chinese medicine diagnostic models have been reported, and they are limited in application to a single disease-type.

**Objective:**

The objective was to develop an artificial intelligence–based assistive diagnostic system capable of diagnosing multiple types of diseases that are common in traditional Chinese medicine, given a patient’s electronic health record notes. The system was designed to simultaneously diagnose the disease and produce a list of corresponding syndromes.

**Methods:**

Unstructured freestyle electronic health record notes were processed by natural language processing techniques to extract clinical information such as signs and symptoms which were represented by named entities. Natural language processing used a recurrent neural network model called bidirectional long short-term memory network–conditional random forest. A convolutional neural network was then used to predict the disease-type out of 187 diseases in traditional Chinese medicine. A novel traditional Chinese medicine syndrome prediction method—an integrated learning model—was used to produce a corresponding list of probable syndromes. By following a majority-rule voting method, the integrated learning model for syndrome prediction can take advantage of four existing prediction methods (back propagation, random forest, extreme gradient boosting, and support vector classifier) while avoiding their respective weaknesses which resulted in a consistently high prediction accuracy.

**Results:**

A data set consisting of 22,984 electronic health records from Guanganmen Hospital of the China Academy of Chinese Medical Sciences that were collected between January 1, 2017 and September 7, 2018 was used. The data set contained a total of 187 diseases that are commonly diagnosed in traditional Chinese medicine. The diagnostic system was designed to be able to detect any one of the 187 disease-types. The data set was partitioned into a training set, a validation set, and a testing set in a ratio of 8:1:1. Test results suggested that the proposed system had a good diagnostic accuracy and a strong capability for generalization. The disease-type prediction accuracies of the top one, top three, and top five were 80.5%, 91.6%, and 94.2%, respectively.

**Conclusions:**

The main contributions of the artificial intelligence–based traditional Chinese medicine assistive diagnostic system proposed in this paper are that 187 commonly known traditional Chinese medicine diseases can be diagnosed and a novel prediction method called an integrated learning model is demonstrated. This new prediction method outperformed all four existing methods in our preliminary experimental results. With further improvement of the algorithms and the availability of additional electronic health record data, it is expected that a wider range of traditional Chinese medicine disease-types could be diagnosed and that better diagnostic accuracies could be achieved.

## Introduction

The field of machine learning has experienced unprecedented and rapid development in recent years; this growth can be attributed to three factors—advanced artificial neural network architecture and algorithms, enhanced computing power, and the availability of vast amounts of training data. Machine learning has been successfully applied to many fields including medical health systems. Applications of machine learning in medical health systems can be roughly classified into two categories—image-based such as radio imaging analysis and text-based such as electronic health record analysis using natural language processing. Numerous reports have shown strong performance of image-based machine learning applications [1-6] while the successful development of text-based medical applications [7,8] remains a challenge because of its unstructured and diverse form of input data. In this age of digital medicine (and its associated deluge of digital information), it has become a daunting task for medical experts to fully utilize medical history and the test result data in a timely fashion; therefore, it is not only possible but necessary that machine learning is used to assist medical professionals in diagnostic and treatment decision making. Systems that can be used to assist medical professionals in this decision making are often called assistive diagnostic systems.

Assistive diagnostic systems have become an intense research focus for both medical practitioners and scientists in the past decade. A typical assistive diagnostic system consists of a functionality that extracts critical clinical information such as symptoms from electronic health record, and another functionality that performs deductive reasoning to predict or diagnose biomedical diseases based upon the extracted clinical information. Liang et al [9] reported an artificial intelligence–based pediatric disease diagnostic system that demonstrated high diagnostic accuracies in diagnosing common childhood diseases across multiple organ systems which was comparable to that of experienced physicians. This was accomplished by using a natural language processing technique to extract relevant symptom information from electronic health record notes, and by using logistic regression classifiers to predict the disease based upon the symptoms.

In comparison to treatment decision making in Western medicine, treatment decision making in traditional Chinese medicine is more challenging. In traditional Chinese medicine, physicians need to perform syndrome differentiation [10] as well as disease diagnosis before a decision concerning treatment can be made. A syndrome is a concept unique to traditional Chinese medicine and is an abstraction of a variety of symptoms and signs—it is a pathological summarization of a specific stage of a disease. A syndrome covers disease location, etiology, and the struggle between the disease’s pathogenic factors and the body’s resistance. In traditional Chinese medicine, the relationship between disease and syndrome is not one-to-one. Instead, disease to syndrome mapping may be considered many-to-many; therefore, the application of machine learning to decision-making processes in traditional Chinese medicine is challenging. Numerous attempts have been made to apply machine learning to traditional Chinese medicine to assist physicians in their treatment decisions [10-13]. Zhou et al [12] proposed a traditional Chinese medicine diagnostic model with multilabel classification. The model takes symptoms as input and predicts medicine disease-type and corresponding syndromes and was able to show good diagnostic accuracy. Liu et al [13] used a deep learning technique and one-versus-the-rest strategy for multilabel classification in diagnostic modeling for syndrome differentiation of traditional Chinese medicine chronic gastritis diseases and also achieved good results.

Despite these encouraging preliminary results, existing artificial intelligence–based traditional Chinese medicine systems have been limited in what their diagnostic model can diagnose (typically only one type of traditional Chinese medicine disease). In practice, it is highly desirable for an assistive diagnostic system to be capable of diagnosing or differentiating between multiple diseases and syndromes.

In this paper, we present an artificial intelligence–based traditional Chinese medicine diagnostic system which can diagnose 187 diseases common in traditional Chinese medicine and predict their associated syndromes from unstructured freestyle electronic health records. In the system, notes from freestyle electronic health record are first processed using a bidirectional long short-term memory network with conditional random forest [14-17] to form structured data, then features are extracted from the structured data and further vectorized. A convolutional neural network for processing text [18,19] was used to predict which traditional Chinese medicine disease was diagnosed from the vectorized data, and an integrated learning model was used to predict the disease’s corresponding syndromes.

## Methods

### Overview

**Figure 1:**
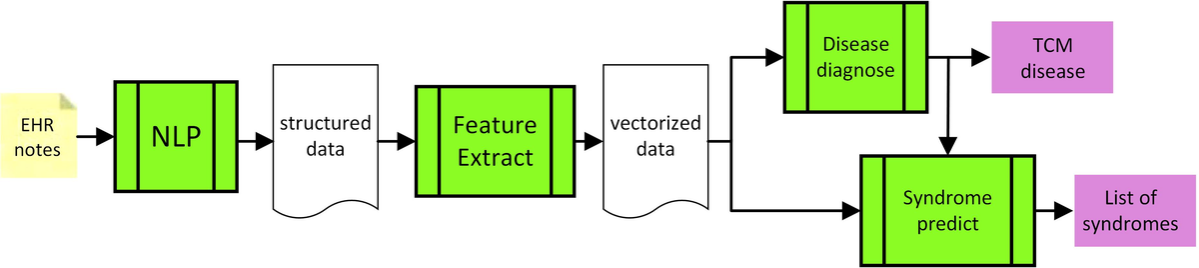
Block diagram of the proposed assistive diagnostic system. EHR: electronic health record; NLP: natural language processing; TCM: traditional Chinese medicine.

A high-level block diagram of the diagnostic system is shown in Figure 1. The system consists of four subsystems—natural language processing, feature extraction, disease diagnosis, and the syndrome prediction. The natural language processing subsystem takes notes from freestyle electronic health record as input, extracts named entities, and produces structured data from the recognized named entities and the relationships among the named entities. The feature extraction subsystem extracts clinical information useful in disease diagnosis and syndrome differentiation from the structured data and produces additional vectorized data as output. The vectorized data are fed into a disease diagnosis network to predict the disease and are then given to the syndrome prediction subsystem to produce a list of syndromes. The syndrome prediction subsystem consists of 187 models, each of which corresponds to a disease in traditional Chinese medicine. The output of the disease diagnosis subsystem is used as input for the syndrome prediction subsystem in order for the syndrome prediction subsystem to select the appropriate model to use.

### Natural Language Processing Subsystem

**Figure 2:**
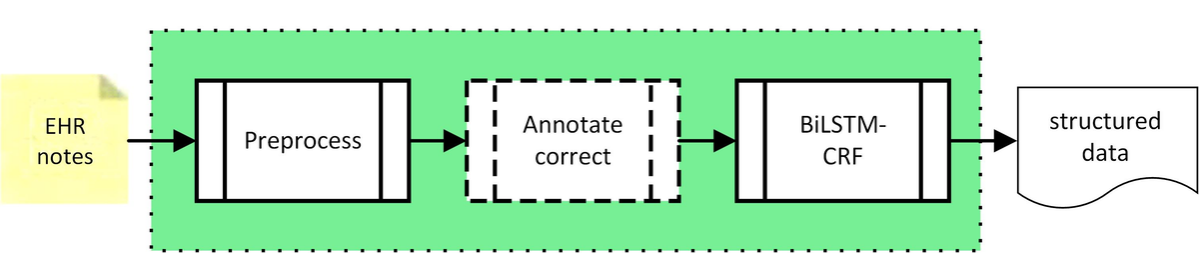
Natural language processing subsystem block diagram. Dashed-lines indicate that the component’s existence is conditional based upon whether the system is in the training phase. BiLSTM-CRF: bidirectional long short-term memory network with conditional random forest; EHR: electronic health record; NLP: natural language processing.

The natural language processing subsystem is responsible for generating structured data from unstructured electronic health record notes. Its internal block diagram is shown in Figure 2. There are three functional blocks in this subsystem. The first block preprocesses electronic health record notes, the second block annotates and corrects, and the third, which is a bidirectional long short-term memory network with conditional random forest, is responsible for named entity recognition. The second block exists only during the training phase of the system; during the testing and application phase, notes do not need to be annotated, thus the second block is bypassed.

#### Electronic Health Record Notes Preprocessing

Electronic health record notes were preprocessed by removing unnecessary or unusable components of the electronic health record such as pictures. Notes were transformed into a standard format (half-angle encoding was used); notes were written in Chinese, and since Chinese characters can be encoded in either full-angle or half-angle format, a standard format was required. Freestyle notes were sorted and divided according to predefined sections such as chief complaint, family medical history, etc.

#### Electronic Health Record Notes Annotation

**Figure 3:**
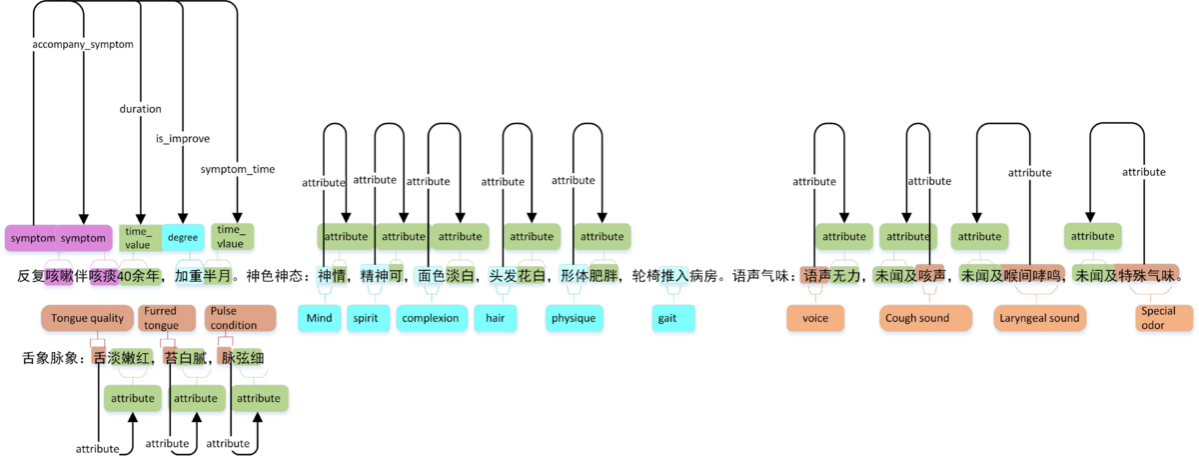
Example of electronic health record notes annotation.

In the training data set, all electronic health record notes were annotated to be used for supervised training of the bidirectional long short-term memory network with conditional random forest, the convolutional neural network for processing text, and the integrated learning model network. Notes were annotated with named entities and the relationships among entities. Figure 3 shows sample annotation of the electronic patient record of a patient with a coughing history of 40 years who experienced severe coughing in the 15 days prior to visiting the physician. The electronic health record indicates that the patient entered the hospital in a wheelchair and was observed as being pale, weak, and in good spirit. Observations based upon physical examination of the tongue were recorded in the notes; tongue quality was observed as being pale red, furred, with white and greasy coating. Through annotation, the notes were marked with named entities such as cough, tongue quality, and pulse observation. Clinical information contained in the electronic health record notes was first processed by computer to form the initial training data. Subsequently, medical experts manually examined and corrected the preliminary results to form the final training data set.

#### Bidirectional Long Short-Term Memory Network With Conditional Random Forest Network for Named Entity Recognition

**Figure 4:**
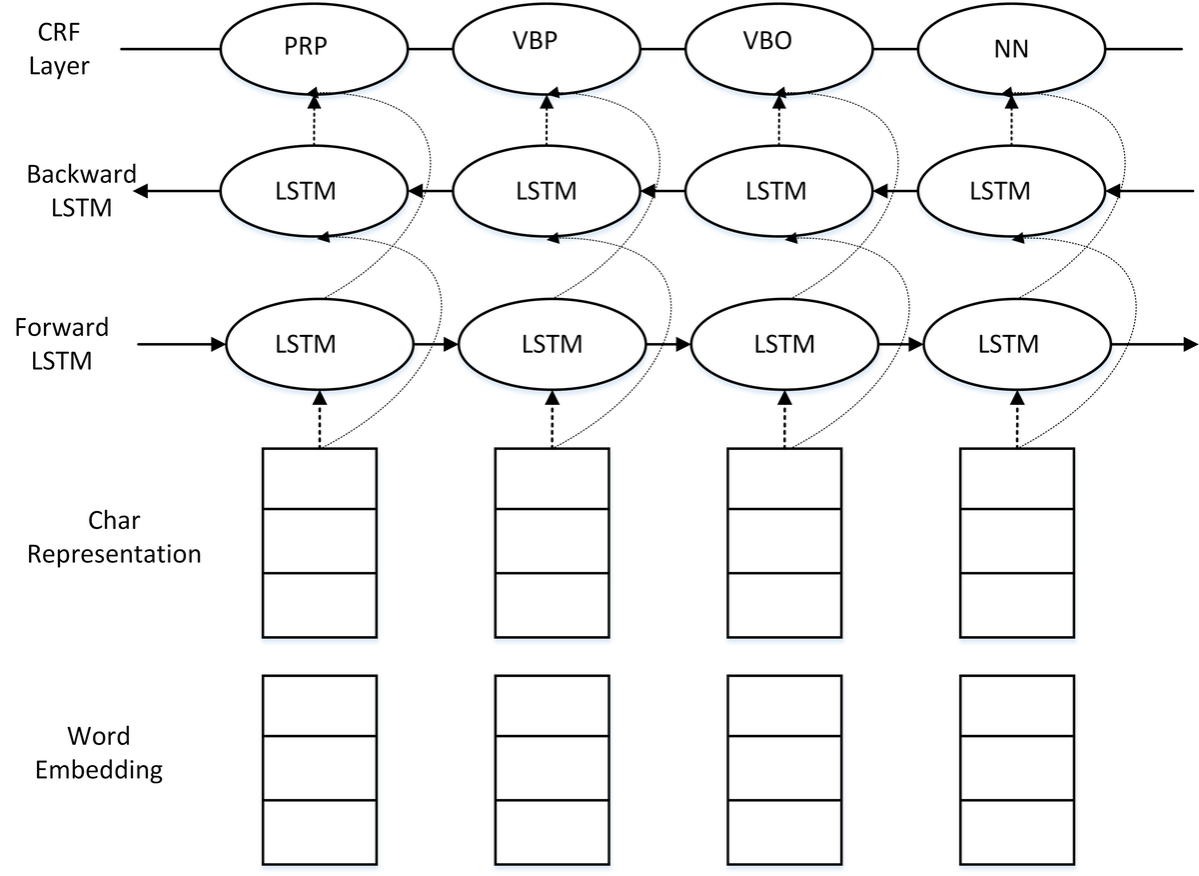
Bidirectional long short-term memory network with conditional random forest block diagram. CRF: conditional random forest; LSTM: long short-term memory; NN: noun, singular speech tag; PRP: personal pronoun speech tag; VBO: verb speech tag; VBP: verb, singular present speech tag.

The bidirectional long short-term memory network with conditional random forest was responsible for generating structured data from the preprocessed electronic health record notes. This was accomplished by employing a recurrent neural network as shown in Figure 4. Numerous studies have shown that a bidirectional long short-term memory network with conditional random forest is best suited for processing sequential data such as speech and text [14-16].The open-source implementation [17] of the model presented by Lample et al [16] was adopted for the construction of our bidirectional long short-term memory network with conditional random forest system.

**Figure 5:**
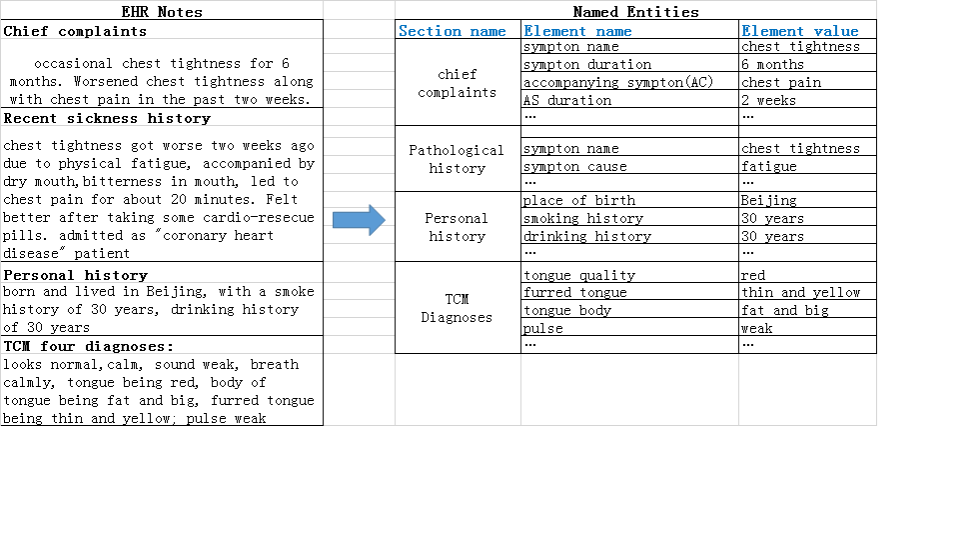
An example of named entity extraction from electronic health record notes. EHR: electronic health record; TCM: traditional Chinese medicine.

With this network, named entities in the electronic health record notes can be extracted and properly placed in predefined data structures according to the relationships among the named entities. Figure 5 shows an example of the mapping between the electronic health record notes and the structured text with predefined sections.

### Feature Extraction Subsystem

**Figure 6:**
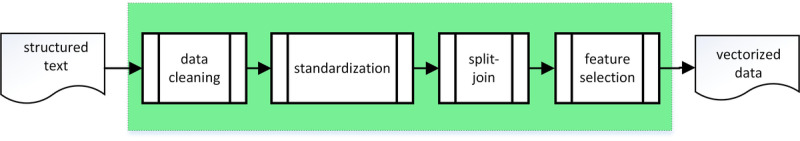
Block diagram of the feature extraction subsystem.

The feature extraction subsystem was responsible for extracting useful information from the structured text data. The internal blocks of this subsystem are shown in Figure 6. The structured text contained many redundant and nonrelevant entries since the data structure was defined for a generalized purpose. During the feature extraction process data were cleaned, descriptions of symptoms and physical conditions were standardized (given that different physicians may have used different wording) using a predefined dictionary, a process called split-and-join was performed. Since the same named entity could be found in different sections of the structured text, for example, in the medical history as well as in the chief complaint, this step split the sections into parts and joined the relevant parts based upon their features.

During feature selection, entities corresponding to the same symptom were correspondingly ordered. For example, a chief complaint of “coughing for 3 days accompanied coughing phlegm for 2 days” contained two symptom entities—“coughing” and “coughing phlegm”—and two time entities—“3 days” and “2 days.” In this example, a total of four symptoms were obtained— “coughing”, “coughing for 3 days”, “coughing phlegm,” and “coughing phlegm for 2 days”— from which a weighted sum was calculated. An entity’s weight was calculated based upon the time distance from the current time as *weight(n+1)* = *weight(n)* + *increment*, where *n*=0, 1, 2, …*N*, *increment*= 1/*N*, *weight(0)*=0 and *N* was the total number of time units. This formula gives a larger weight value to an entity that is nearer in time to the current time, and a smaller weight value to an entity that is further in the past. The weighted sum was used to decide which features were extracted and the extracted features were output as vectorized data.

### Convolutional Neural Network for Processing Text Disease Diagnosis Subsystem

Convolutional neural networks are composed of alternating convolution and pooling layers and a fully connected layer. Due to the characteristics of convolution kernel, the features represented by adjacent elements in a 2-dimensional space can be mined. Similarly, in the field of natural language processing field, 1-dimensional convolution kernels can be used to mine correlations among different words in a sentence. A network that uses convolutional neural networks for natural language text processing is called a convolutional neural network for processing text network. After word segmentation of a Chinese sequence, word embedding represented each word with a high-dimensional vector denoted by floating-point numbers to convert a sentence into a 2-dimensional matrix. Convolution operations are performed on the 2-dimensional matrix with multiple convolution kernels whose widths were equal to the dimension of the word vector dimension but which were of different heights. Pooling operations were then performed to classify and to predict Chinese text [19].

**Figure 7:**
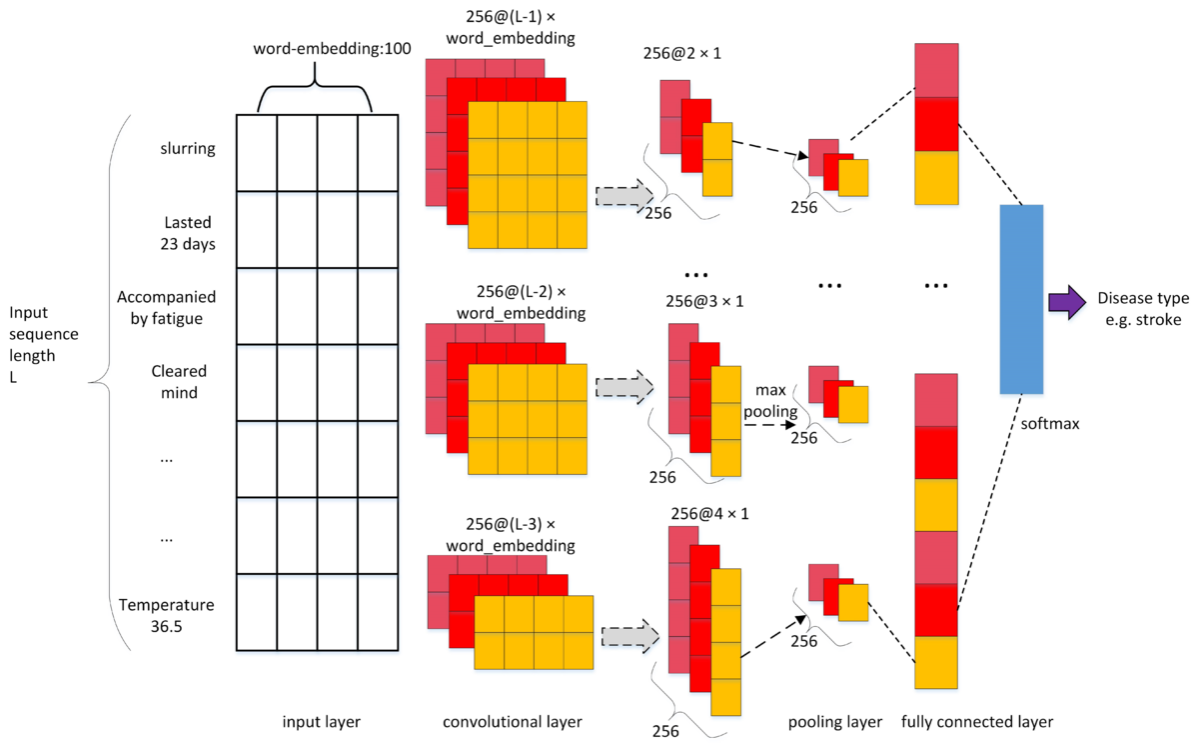
Illustration of the text convolutional neural network.

The structure of the convolutional neural network model used in this study is shown in Figure 7. The inputs were the named entities and their relationships that were extracted from the database of the structured medical record information. To ensure that the input length was consistent, the maximum number of words in the sample was set to *L* and zero-padding was used. From the word embedding layer, a word matrix with a size of 149,076×100 was obtained. The word vector model used in this experiment was trained by the public open-source Gensim module, whose corpus is composed of Chinese electronic health record data from multiple hospitals. The dictionary contained 149,076 words, each word represented by a 100-dimensional word vector. A 2-dimensional convolution was used in this convolutional neural network. When selecting the model structure and parameters, various factors such as sample size, hardware equipment performance, model complexity, characteristics of the electronic health records, and past experimental experience were considered. The grid search method was used to set multiple values in descending order, and to select the best parameter value from different ranges and magnitudes of the same parameter. By comparing the accuracy of the trained model with that of the test set, we set 256 convolution kernels with dimensions of (*L*-1)*100, (*L*-2)*100, and (*L*-3)*100 (as filters), from which 256 2×1, 3×1, and 4×1 feature surfaces were obtained. A maximum pooling layer was added to perform dimension reduction on the features of the filter layer. Finally, the pooled vectors were stitched through the fully connection layer as the input of the softmax (normalized exponential) layer to predict the disease from 187 possible classes. Due to the complexity of the multiclass classification problem and because the input electronic health record data may not be ideal, the prediction accuracy of the model in the first class cannot reach accuracy of 100%. The top five classes can be predicted accurately and also have practical significance; therefore, the top five are used in the model prediction as the final output.

### Integrated Model for Syndrome Prediction Subsystem

Syndrome differentiation is an integral part of treatment in traditional Chinese medicine. The syndrome prediction subsystem produces a list of the most probable syndromes based upon the structured vector data. In theory, many machine learning algorithms could be used for syndrome prediction; however, in practice, not all are suited to the task due to the characteristics of the relationships between disease and its associated syndromes in traditional Chinese medicine. For example, text processing convolutional networks were ruled out since they cannot perform well in a situation where the number of syndromes associated with a disease is small. Back propagation [20] neural networks have strong nonlinear mapping capabilities because they can approach arbitrarily close to any continuous curve. Furthermore, back propagation possesses flexibility in terms of the number of network layers, the number of neurons in a layer, and the learning rate coefficients. Thus, back propagation networks have been favored in traditional Chinese medicine modeling [20]. The support vector classifier [21] has a strong mathematical basis and has shown excellent performance in situations where the number of samples is small, the dimension is high, and there is strong nonlinearity which is why support vector classifiers have previously been used for syndrome prediction. Random forest [22] models have also been used for syndrome prediction. Random forests employ bootstrap aggregation ensemble methods which combine the predictions from multiple independent decision trees. Extreme gradient boosting [23] has recently become popular and has proven to be effective in syndrome prediction.

**Figure 8:**
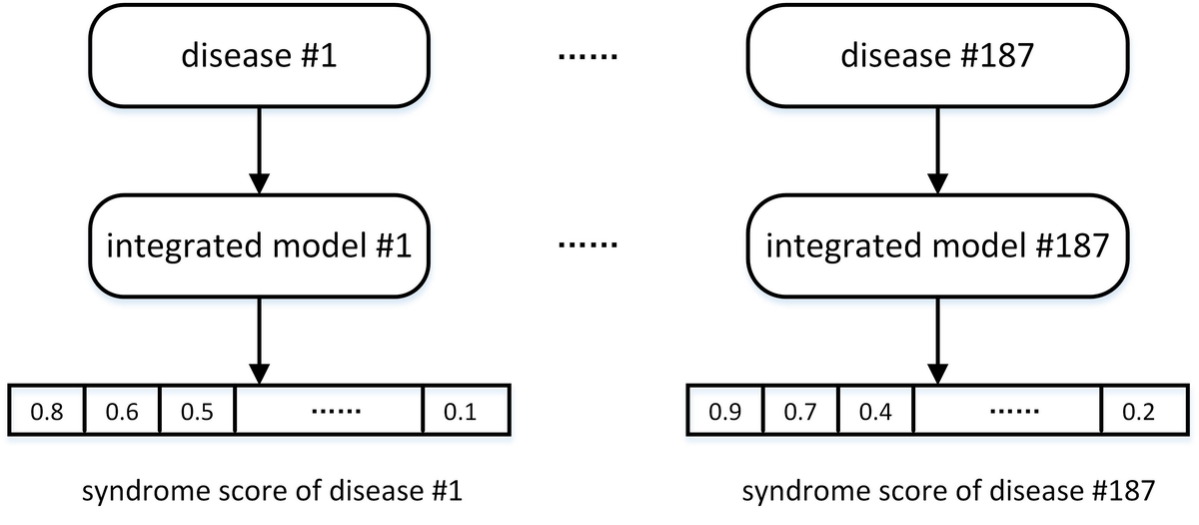
Illustration of the 187 integrated models.

A closer examination of these four algorithms for syndrome predictions demonstrated that, individually, they are prone to either underfitting or overfitting in applications of syndrome prediction. In our system, they were collectively employed to form an integrated learning model for a given disease-type. In this integrated learning model, the bootstrap aggregation (random forest) ensemble method was used to combine the predictions from different methods such as back propagation and support vector classifier. The extreme gradient boosting was used to combine weak classifiers into a strong classifier. Figure 8 illustrates the 187 integrated models, each of which can produce a list of syndromes for a given disease.

**Figure 9:**
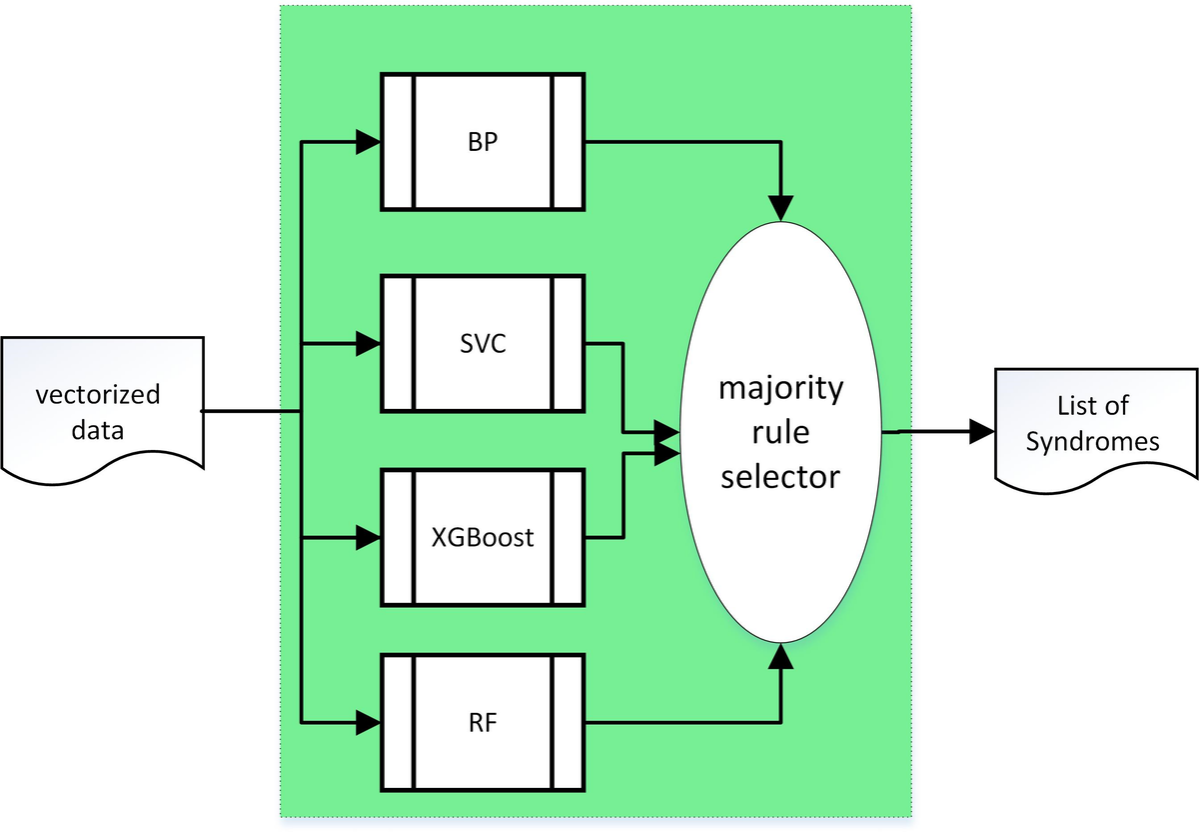
Block diagram of the Integrated learning model. BP: back propagation; RF: random forest; SVC: support vector classifier; XGBoost: extreme gradient boosting.

Each of the models consists of four individual algorithms—back propagation, support vector classifier, random forest, and extreme gradient boosting. As shown in Figure 9, the integrated model selects the final output from the outputs of the four algorithms by majority-rule. In *majority-rule*, the selection decision is based upon the highest number of votes for the outputs from each of the four algorithms. This approach not only overcomes the drawbacks of underfitting and overfitting, but also takes advantage of the strength of individual algorithms in predicting syndromes for some but not other types of diseases.

The integrated learning model approach has a better capability for generalization compared to the capabilities of existing approaches. This allows our artificial intelligence–based assistive diagnostic system to handle 187 classes of disease while existing systems may only be capable of handling one or two.

## Results

### Data Source

The data set used in this research was obtained from Guanganmen traditional Chinese medicine Hospital in Beijing, China. A total of 22,984 electronic health record notes that were generated between January 1, 2017 and September 7, 2019 were used for the training, validation, and testing of this system. This data set contained 187 first-category diseases and a total of 466 first-category syndromes. Furthermore, these 187 traditional Chinese medicine diseases are among the 236 most common diseases in traditional Chinese medicine [24]. These diseases cover internal medicine, gynecology, pediatric, orthopedics and traumatology, otolaryngology, dermatology, and surgery.

Originally, there were 23,719 electronic health record notes. A quality control process was applied to exclude notes with incomplete records such as missing admission page or discharge page, or notes with inconsistent information such as conflicting information between admission page and discharge page. In addition, notes that did not contain standard descriptions of complaint were discarded to eliminate biased or incorrect opinions from different physicians. From this process, 735 notes were discarded resulting in a total of 22,984 notes that were included.

The distribution of the number of electronic health record notes for different diseases and syndromes was 2180, 1913, 109, and 584 notes for cluster disease, diabetes, asthma, and spleen disease, respectively. Since this distribution imbalance would lead to bias that favors those with a large number of training samples and would reduce the system’s generalization capability, to mitigate this issue, upsampling and downsampling were used to preprocess the original data set in order to make sample distribution approximately even. Upsampling with the synthetic minority oversampling technique was used to increase the number of electronic health record notes for asthma and spleen disease to 1000 each, while the number of electronic health record notes for cluster disease and diabetes were each trimmed to 1000 through downsampling.

The processed data set was then partitioned into the training set, the validation set, and the testing set in a typical ratio of 8:1:1. The training set was used to train the coefficients of the models, the validation set was used for adjusting the model parameters, and the test set was used for measuring the performance of the system. During the partition of the data set, a k-fold+bootstrap resampling technique was employed to process the training set and the validation set. Generalization capability was improved by searching the best superparameters on different partitions during the training and integration of multiple models.

### Validation

One-tenth of the total number (2298/22,984) of electronic health record in the data set was used for validation. The purpose of validation was to fine tune the neural network parameters.

### Disease Diagnosis Results

The convolutional neural network for processing text disease diagnostic system was trained with a data set that contains 187 types of traditional Chinese medicine diseases. The test data set contained 2298 copies of electronic health record notes. The test results on the trained convolutional neural network for processing text model were 83.9% for the top 1 score, 92.4% for the top 3 score, and 95.7% for the top 5 score. As indicated by the test results, disease diagnosis generated relatively high diagnostic accuracies for the top 1, top 3, and top 5 score. The top 1 score, the top 3 score, and the top 5 score were calculated as follows. First, the list of predicted diseases was sorted into descending order based upon associated probabilities. If the number one predicted disease matched the target disease, then the test was considered a success for the top 1 score. If one of the first three predicted diseases matched the target disease, then the test was considered a success for the top 3 score. If one of the first five predicted diseases matched the target disease, then the test was considered a success for the top 5 score.

Based on the above definitions for the top 1 score, the top 3 score, and the top 5 score, naturally, it is always valid that the top 5 score > the top 3 score > the top 1 score. The results also suggest that even for only the top 1 score, prediction accuracy is high.

### Syndrome Prediction Results

For each traditional Chinese medicine disease, a syndrome prediction model of that disease was trained. Under the same experimental conditions and data set, a 5-fold cross-validation method was used to evaluate the prediction results of each of the four algorithms and that of the integrated learning model. The calculated prediction accuracies of all 187 traditional Chinese medicine diseases were calculated. For the sake of brevity, only the results of 12 disease were included in Table 1 along with the average accuracies over all 187 diseases.

As shown in Table 1, extreme gradient boosting generally outperformed back propagation, support vector classifier, and random forest methods. Furthermore, the integration learning model reached an average prediction accuracy of 0.91, better than any of the four known models. The reason for the outstanding performance of the integration model lies in the fact that it employed a majority-rule selection method for the final prediction result.

**Table 1:** Syndrome prediction results.

Syndrome		Model Accuracy				
		Back Propagation	Support Vector Classifier	Random Forest	Extreme gradient boosting	Integration
**Medical condition**						
	Bloody Stool	0.872	0.868	0.886	0.890	0.942
	Abdominal Pain	0.808	0.800	0.822	0.880	0.877
	Cough	0.832	0.872	0.894	0.896	0.935
	Stroke	0.823	0.852	0.892	0.903	0.912
	Insomnia	0.841	0.925	0.986	0.950	0.984
	Hemoptysis	0.802	0.799	0.932	0.919	0.972
	Blindness	0.784	0.779	0.790	0.846	0.803
	Depression	0.809	0.817	0.819	0.820	0.826
	Asthma	0.953	0.879	0.951	0.950	0.954
	Anorectal Disease	0.908	0.861	0.879	0.901	0.893
	Pepey Disease	0.832	0.865	0.870	0.887	0.892
Mean accuracy (of all 187)		0.822	0.872	0.868	0.886	0.913

### Assistive Traditional Chinese Medicine Diagnostic System

Existing assistive traditional Chinese medicine diagnostic systems that have been reported can handle only one type of disease or a few syndrome predictions. Our system can diagnose 187 traditional Chinese medicine diseases and the associated syndromes. So far, we have not found systems similar to ours.

The overall system-level prediction accuracy was calculated by dividing the total number of correct predictions by the number of test cases. A correct prediction was defined by both disease and syndrome having been correctly predicted, simultaneously.

The test results of system-level accuracy were 80.5% for the top 1 score, 91.6% for the top 3 score, and 94.2% for the top 5 score. Our disease diagnosis model and syndrome prediction model together yielded relatively high diagnostic accuracies for the top 1 score, top 3 score, and top 5 score.

## Discussion

### Principal Results

Unlike most previous research projects which have typically focused on traditional Chinese medicine syndrome prediction for only one disease-type, we successfully used machine learning to simulate hypothetical deductive reasoning similar to that of human physicians in order to diagnose traditional Chinese medicine disease and corresponding syndromes for 187 types of diseases. The state-of-art syndrome prediction accuracy was obtained by employing a new syndrome prediction model. The prediction accuracy of this system is sufficient to assist traditional Chinese medicine practitioners in their daily clinical work.

### Limitations

The data set, which only spanned two years in a single traditional Chinese medicine hospital, was relatively small. Not all common diseases and syndromes were contained in this data set; therefore, additional clinical data are needed to further improve the system.

### Comparison With Prior Work

Prior work reported by other researchers mainly focused on one particular type of traditional Chinese medicine disease [20-23]. Our work is centered around the capability of diagnosing all the traditional Chinese medicine diseases and associated syndromes. At present, the proposed system can diagnose 187 out of 236 common traditional Chinese medicine diseases.

### Conclusions

Artificial intelligence in diagnosing patients is highly desirable in today’s digital medical age. With an abundance of medical information contained in freestyle medical health record notes, machine learning–based assistive systems can mine the medical data to extract useful and logical information and form preliminary opinions on diseases and treatment plans. For traditional Chinese medicine, syndrome prediction is also part of the diagnostic process. Because traditional Chinese medicine diseases can be linked to many syndromes, and syndromes can be linked to many diseases, disease diagnosis and syndrome prediction are more challenging in traditional Chinese medicine than in Western medicine. An effective artificial intelligence–based traditional Chinese medicine assistive diagnostic system was developed in this research by employing bidirectional long short-term memory network with conditional random forest for named entity recognition, a convolutional network for text processing for disease diagnosis, and an integrated learning model for syndrome prediction. The main contribution of this paper is a novel syndrome prediction scheme and convolutional network that represents 187 traditional Chinese medicine diseases. The system was trained, validated, and tested by using the data set obtained from nearly 23,000 electronic health record notes from Guanganmen Hospital. The proposed system distinguishes itself from existing assistive systems in that it can predict traditional Chinese medicine disease-type and syndromes simultaneously, and it can diagnose 187 types of common traditional Chinese medicine diseases. Furthermore, preliminary results suggest that the system achieved higher prediction accuracy than all existing systems [20-23]. Future work will include optimizing the convolutional network for processing text to learn all 236 common traditional Chinese medicine diseases, further improvement of the integrated learning model for syndrome prediction, and the use of additional electronic health record notes to train the system.

## Abbreviations

BiLSTM-CRF: bidirectional long short-term memory network with conditional random forest
CRF: conditional random forest
EHR: electronic health record
LSTM: long short-term memory
NN: noun, singular speech tag
PRP: personal pronoun speech tag
VBO: verb speech tag
VBP: verb, singular present speech tag
NLP: natural language processing
